# Cardioplegia practice in paediatric cardiac surgery: a UK & Ireland survey

**DOI:** 10.1177/0267659118794343

**Published:** 2018-08-10

**Authors:** Nigel E. Drury, Angela Horsburgh, Rehana Bi, Robert G. Willetts, Timothy J. Jones

**Affiliations:** 1 Department of Paediatric Cardiac Surgery, Birmingham Children’s Hospital, Birmingham, UK; 2 Institute of Cardiovascular Sciences, University of Birmingham, Birmingham, UK

**Keywords:** cardioplegia, myocardial protection, paediatric cardiac surgery

## Abstract

**Introduction:**

Many techniques are available for cardioplegic arrest in children, but there is a lack of late phase clinical trials to guide practice. We surveyed paediatric cardiac surgeons and perfusionists to establish current practice and willingness to change within a clinical trial.

**Methods:**

An online survey was sent to all consultant paediatric cardiac surgeons and chief perfusionists in paediatric centres in the UK and Ireland. Information was sought on cardioplegia type, composition, temperature, topical cooling, dosing for induction and maintenance, interval between doses, whether practice changed with patient age or complexity and whether respondents would be willing and able to use different cardioplegia solutions within a randomised trial.

**Results:**

Responses were obtained from 32 (78.0%) surgeons and 12 (100%) perfusionists. Twenty-seven (84.4%) surgeons use blood cardioplegia in infants, with St. Thomas’ Harefield preparation the most popular (19, 59.4%), used routinely in eight (66.7%) centres. Twenty-two (68.8%) administer at 4-6°C, 18 (56.3%) use topical cooling, 18 (56.3%) give 30 ml/kg induction and 15 ml/kg maintenance, with 23 (71.9%) re-dosing every 20-25 minutes. Thirty (93.8%) surgeons were open to randomising patients in a trial, with del Nido (29, 90.6%) the most popular.

**Conclusions:**

This survey demonstrates heterogeneity in cardioplegia practice. Whilst most surgeons use blood cardioplegia, there is variation in type, temperature, topical cooling, dosing and intervals. Combined with a lack of evidence from late phase trials, our findings support the presence of clinical equipoise. Surgeons are willing to change practice, suggesting that a pragmatic, multi-centre, randomised, controlled trial of cardioplegia in children is feasible.

## Introduction

Cardioplegia is fundamental to the intracardiac repair of congenital heart defects. In combination with hypothermia, it remains the mainstay of myocardial protection against ischaemia-reperfusion injury during cardiac surgery, providing access to a still and bloodless field through electromechanical arrest.^1^ However, the ubiquitous release of troponin following aortic cross-clamping in children demonstrates that myocardial injury is a routine occurrence.^2,3^ Low cardiac output syndrome remains the most common premorbid complication after cardiac surgery in children and the most frequent seminal event leading to death.^4^ As current cardioplegia techniques were mostly derived from adult or laboratory models, they may not provide optimal protection for the immature myocardium.^5–7^

Recent surveys of practice have shown marked variations in the use of commercially-available and customised cardioplegia solutions in children in North America^8^ and worldwide.^9^ This may be driven by a lack of evidence to support one technique over another; the paediatric cardiac surgery literature contains no late phase, randomised, controlled trials and there is a need for high-quality evidence to guide practice and improve outcomes.^10^ We, therefore, conducted a survey to establish the current cardioplegia practice in paediatric cardiac surgery in the UK & Ireland and the willingness of surgeons to randomise patients to different solutions within the context of a multi-centre, randomised, controlled trial.

## Methods

A survey link was sent via email to all 41 consultant paediatric cardiac surgeons and the chief perfusionist (identified via The Society of Clinical Perfusion Scientists) at each of the 12 centres performing children’s heart surgery in the United Kingdom and the Republic of Ireland in December 2017. Non-responders received a second personalised follow-up email in January 2018 to prompt completion. Respondents were required to provide consent for anonymous reporting of the information provided. The survey data were collected and managed using the REDCap electronic data capture tools run by the Birmingham Surgical Trials Consortium at the University of Birmingham, under licence from Vanderbilt University.^11^

The study design was informed by the Congenital Heart Surgeons’ Society survey of Kotani et al.^8^ A full list of questions is available as supplementary material. In brief, data was requested from each respondent for their current/usual cardioplegia practice in infants (30 days-1 year), including type of solution, composition, temperature, topical cooling, dosing for induction and maintenance and interval between doses. Respondents were asked whether their practice differs by patient age (neonates, children over 1 year) and expected complexity of the repair and to provide details. Perfusionists were also asked for details of their cardioplegia-delivery system and whether any additional equipment or disposables would be required for other cardioplegia types.

Finally, respondents were asked whether they would be willing to use different types of cardioplegia within the context of a clinical trial, specifically: del Nido cardioplegia, as used at Boston Children’s Hospital;^12^ Custodiol HTK (Dr Franz Köhler Chemie, Bensheim, Germany); St. Thomas’ blood Harefield preparation (Terumo BCT, Larne, UK); or St. Thomas’ Hospital crystalloid solution No. 2 (Plegisol, Abbott Laboratories, Chicago, IL). If so, they were asked whether there are any patient groups in whom they would not be willing to randomise to each solution and what interval between doses would be acceptable, assuming no electromechanical activity; if not, they were asked to describe their concerns over using that solution.

Statistical analysis was performed using Excel (Microsoft Corporation, Redmond, WA). Categorical data were expressed as counts and percentages. The corresponding author had full access to all the data in the study and had final responsibility for the decision to submit for publication.

## Results

The survey was completed by 44 of 52 (84.6%) healthcare professionals contacted, 32 of 41 (78.0%) consultant surgeons and all 12 (100%) chief perfusionists.

**Figure 1.**
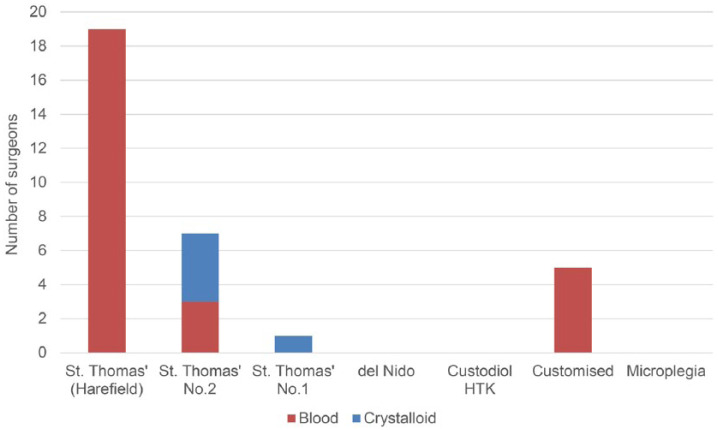
Types of cardioplegia solution used in infants.

Of the 32 surgeons who responded, 27 (84.4%) use blood cardioplegia in infants, with St. Thomas’ Harefield preparation in a 4:1 blood-to-crystalloid ratio being the most popular (19, 59.4%) and used routinely in eight (66.7%) centres (Figure 1). Five (15.6%) surgeons from two centres use bespoke institutional depolarising blood cardioplegia solutions; no centres currently use del Nido solution as it has not been available commercially in the UK or Ireland. Two (6.3%) surgeons who use blood cardioplegia in older children switch to crystalloid cardioplegia in neonates; conversely, three (9.4%) surgeons who use crystalloid in infants change to blood in older children. No surgeons make significant changes to their cardioplegia strategy depending on the expected complexity of the repair alone.

Variations in the techniques of surgeons responding to the survey are shown in Table 1. The preferred temperature of administration is 4-6°C for 22 (68.8%) surgeons, whether using a blood or crystalloid solution; additional topical cooling of the heart is employed routinely by 14 (43.8%) and selectively by four (12.5%), with only one (3.1%) considering giving a warm terminal ‘hot shot’ dose in long cross-clamp times. All surgeons administer antegrade cardioplegia, with two (6.3%) using a combined antegrade and retrograde approach in selected cases. The majority (18, 56.3%) give an induction dose of 30 ml/kg and maintenance of 15 ml/kg, with six (18.8%) opting for an induction of 20-25 ml/kg and maintenance of 10 ml/kg. The usual/preferred interval between doses is every 20-25 minutes for 23 (71.9%) and 30-35 minutes for eight (25.0%). The main indication for re-dosing is time elapsed (28, 87.5%), even in the absence of overt electromechanical activity; one surgeon (3.1%) uses a single dose unless cardiac activity becomes disruptive to the repair.

**Table 1.** Variations in the techniques used by responding surgeons (n=32).

Characteristic	n (%)
Temperature of cardioplegia	
<4°C	2 (6.3%)
4-6°C	22 (68.8%)
7-9°C	5 (15.6%)
10-12°C	1 (3.1%)
>12°C	2 (6.3%)
Topical cooling of heart	
Routine	14 (43.8%)
Selective	4 (12.5%)
No	14 (43.8%)
Terminal warm dose ‘hot shot’	
No	31 (96.9%)
Selected cases	1 (3.1%)
Route of delivery	
Antegrade only	30 (93.8%)
Combined in selected cases	2 (6.3%)
Change practice by patient age	
No change	27 (84.4%)
Switch to crystalloid in neonate	2 (6.3%)
Switch to blood in older child	3 (9.4%)
Induction dose	
10 ml/kg	1 (3.1%)
20 ml/kg	7 (21.9%)
25 ml/kg	2 (6.3%)
30 ml/kg	18 (56.3%)
Determined by BSA	4 (12.5%)
Maintenance dose	
⩽10 ml/kg	2 (6.3%)
15 ml/kg	6 (18.8%)
20 ml/kg	18 (56.3%)
25 ml/kg	2 (6.3%)
Determined by BSA	4 (12.5%)
Indication for maintenance dose	
Time since last dose	28 (87.5%)
Electromechanical activity	1 (3.1%)
Depends on case / other	3 (9.4%)
Interval between doses	
20-25 minutes	23 (71.9%)
30-35 minutes	8 (25.0%)
Usually single dose	1 (3.1%)

BSA: body surface area.

In all centres, cardioplegia is administered by the perfusionist, using a roller pump although, in one centre, crystalloid cardioplegia is given by the anaesthetist, using a handheld syringe/pressure bag, when required. A single-pass system is used by eight (66.7%) centres, recirculating by one (8.3%) and either, depending on the cardioplegia type, by one (8.3%). Perfusionists reported that no additional equipment other than disposables would be required to deliver alternative cardioplegia types at every centre.

Within the context of a clinical trial, 29 (90.6%) surgeons were willing to randomise to del Nido cardioplegia; of these, concerns were raised by five (17.2%) regarding patients with an expected short aortic cross-clamp time, such as atrial or ventricular septal defect repair, by four (13.8%) for use in neonates and by two (6.9%) for those in whom the need to re-clamp the aorta was anticipated. The acceptable interval between doses with del Nido cardioplegia, assuming no electromechanical activity, was up to 60 minutes for 17 (58.6%) surgeons, up to 90 minutes for 10 (34.5%) and up to 120 minutes for one (3.4%), with one not responding. Unsurprisingly, St. Thomas’ Harefield preparation was acceptable to 24 (75.0%) surgeons, most of whom currently use it routinely; two (6.3%) others expressed concern over its use in neonates due to its higher viscosity. St. Thomas’ Hospital crystalloid solution No. 2 (20, 62.5%) and Custodiol HTK (19, 59.3%) were less popular, with concerns raised over the volume of crystalloid required, perceived inferior myocardial protection and that both would constitute a ‘backwards step’ from blood cardioplegia. The greatest combined acceptability was for St. Thomas’ Harefield preparation and del Nido solution, with 24 (75.0%) surgeons from 11 of the 12 centres willing to randomise patients to either option (Table 2). Only two (6.3%) respondents were not open to changing their practice within a trial setting.

**Table 2.** Willingness of responding surgeons to use different types of cardioplegia within a clinical trial (n=32).

Cardioplegia types	n (%)
Individual solutions	
del Nido	29 (90.6%)
St. Thomas’ blood (Harefield preparation)	24 (75.0%)
St. Thomas’ Hospital crystalloid No. 2	20 (62.5%)
Custodiol HTK	19 (59.3%)
Combinations	
del Nido + St. Thomas’ (Harefield)	24 (75.0%)
del Nido + St. Thomas’ crystalloid No. 2	18 (56.3%)
del Nido + Custodiol HTK	18 (56.3%)
St. Thomas’ (Harefield) + St. Thomas crystalloid No. 2	17 (53.1%)
St. Thomas’ (Harefield) + Custodiol HTK	14 (43.8%)
St. Thomas crystalloid No. 2 + Custodiol HTK	13 (40.6%)
Not willing to change practice	2 (6.3%)

HTK: histidine-tryptophan-ketoglutarate.

## Discussion

In this survey of current paediatric cardioplegia practice in the UK and Ireland, we identified variations in practice, although the majority use a similar strategy of antegrade cold blood cardioplegia with a few adjustments for patient age or anticipated length of ischaemia and frequent re-dosing based on time since last dose. However, most surgeons are not devoted to one approach, with widespread willingness to change practice and randomise patients within a clinical trial, perhaps because the optimal solution for paediatric myocardial protection is not established.

Previous surveys of practice have described variations in the application of cardioplegia solutions in children in other healthcare systems. Kotani and colleagues collected data from 56 members of the Congenital Heart Surgeons’ Society in North America and found marked variation in the type of cardioplegia used.^8^ Whilst blood-based cardioplegia was preferred by 86% of respondents, with del Nido solution (38%) the most popular, the next most frequent was ‘other’ (34%), custom-mixed solutions unique to an individual centre. On the other hand, there was consistency in the use of cold (⩽10°C) cardioplegia (93%), an exclusively antegrade route of administration (89%) and, to a lesser extent, topical cooling of the myocardium (64%). Harvey and colleagues surveyed 146 paediatric centres across 5 continents, finding similar variation in the types of cardioplegia used; whilst depolarising solutions were predominant, 32% of North American centres routinely used del Nido solution and 31% of European centres used Custodiol HTK.^9^ Finally, in an earlier survey from Japan, Itoh and colleagues reported that 58% of centres used exclusively crystalloid cardioplegia, 32% used only blood and 10% used both, with 70% giving the cardioplegia at or below 10°C.^13^

Widespread variations in care suggest a lack of definitive evidence to support one technique over another. Randomised, controlled trials represent the gold standard in evaluating healthcare interventions through rigorous testing of a predefined protocol and minimisation of bias.^14^ In a recent systematic review, we identified 26 clinical trials of cardioplegia in paediatric cardiac surgery in the published literature; however, these were exclusively small, single-centre, phase II trials of low value, uncertain quality and at risk of systematic bias.^10^ The heterogeneity of patients, interventions and reported outcome measures across trials also precluded the pooling of results for meta-analysis. Furthermore, these trials included very few neonates, a high-risk cohort in whom myocardial metabolism and cellular homeostasis differ from the more mature heart and the protective effects of cardioplegia are less well understood.^3^

The lack of evidence from late phase clinical trials to support clinical decision-making on cardioplegia in children, combined with the widespread variability in practice demonstrated in this study, suggest clinical equipoise. This is confirmed by the willingness of almost all respondents to change their practice and supports the feasibility of a multi-centre clinical trial. Such a trial would need to be pragmatic, considering the variations in practice and concerns raised in the survey by potential collaborators, to maximise participation and make the findings applicable to routine clinical practice. It would require clinically important endpoints that, if positive, would be convincing to the wider community and, thereby, have the potential to change practice. These should include both validated early outcomes to have contemporary impact and the long-term functional outcomes that are most important to patients and their families.

The strengths of this study include the high response rate and completeness of answers, including surgeons and perfusionists from all centres in the UK and Ireland. Limitations include the applicability of our findings to other countries and the inability to determine whether the variations in practice described have any impact on patient outcomes. Whilst few respondents changed their strategy due to the age of the child or complexity of the repair, the impact of other factors, such as cyanosis or ventricular hypertrophy, on decision-making was not explicitly sought.

In conclusion, this survey of current cardioplegia practice in paediatric cardiac surgery in the UK and Ireland demonstrates heterogeneity in the care provided to children, according to their surgeon and centre. As shown in previous studies,^8,9^ whilst most surgeons now use blood cardioplegia, there is variation in the type of solution, temperature, use of topical cooling, dosing and intervals between doses. In combination with the lack of evidence from late phase clinical trials,^10^ our findings support the presence of clinical equipoise. Indeed, the surgeons’ willingness to change practice within a clinical trial suggests that a pragmatic, multi-centre, randomised, controlled trial of del Nido solution versus St. Thomas’ blood cardioplegia in children is feasible.

## Supplemental Material

PaediatricCardioplegiaSurvey_2018 – Supplemental material for Cardioplegia practice in paediatric cardiac surgery: a UK & Ireland survey Supplemental material, PaediatricCardioplegiaSurvey_2018 for Cardioplegia practice in paediatric cardiac surgery: a UK & Ireland survey by Nigel E. Drury, Angela Horsburgh, Rehana Bi, Robert G. Willetts and Timothy J. Jones in Perfusion
