# Supplementary material for: Cardioplegia practice in paediatric cardiac surgery: a UK & Ireland survey
Source: Perfusion. 2018 Aug 10;34(2):125–9. doi: 10.1177/0267659118794343 (PMC6378396; doi:10.1177/0267659118794343)
Supplement: PaediatricCardioplegiaSurvey_2018 – Supplemental material for Cardioplegia practice in paediatric cardiac surgery: a UK & Ireland survey [file PaediatricCardioplegiaSurvey_2018.pdf]

Survey of paediatric cardioplegia practice 2017/18

We are conducting this survey of current practice in paediatric cardioplegia in the UK & Ireland to identify variations in practice and willingness to change within the context of a proposed multi-centre randomised controlled trial.

This survey will take approximately 10-15 minutes to complete. You may Save & Return Later below then follow the link in the email or click 'Returning?' at top right of this page. Your participation is entirely voluntary and you can withdraw at any time before the survey closes on 31st January 2018 by emailing: nigel.drury@nhs.net

By completing this survey, you give consent that the data you provide can be used anonymously in any arising outputs including publications. Identifiable information is collected only to facilitate future discussions relating to the proposed clinical trial and is strictly confidential.

◯ I give consent

Please enter your name:

____

At which paediatric cardiac surgery centre in the UK or Ireland do you work?

◯ Birmingham - Children's Hospital
◯ Bristol - Royal Hospital for Children
◯ Dublin - Our Lady's Children's Hospital
◯ Glasgow - Royal Hospital for Children
◯ Leeds - General Infirmary
◯ Leicester - Glenfield Hospital
◯ Liverpool - Alder Hey Hospital
◯ London - Evelina London Children's Hospital
◯ London - Great Ormond Street Hospital for Children
◯ London - Royal Brompton Hospital
◯ Newcastle - Freeman Hospital
◯ Southampton - Wessex Cardiothoracic Centre

What is your role?

◯ Consultant Paediatric Cardiac Surgeon
◯ Chief Perfusionist

Who administers cardioplegia in your paediatric practice?

◯ Surgeon
◯ Perfusionist
◯ Anaesthetist
◯ Other

Other - please provide details:

____

How is the cardioplegia given?

◯ Roller pump
◯ Pressure bag
◯ Handheld syringe
◯ Infusion pump
◯ Automated system
◯ Other

Other - please provide details:

____

What type of cardioplegia system do you use?

◯ Recirculating
◯ Single-pass
◯ Syringe injection
◯ Other

Please provide details of your cardioplegia system:

____

How do you measure the volume of cardioplegia given? __________________________________

Please answer the following questions regarding your usual/current practice in INFANTS (30 days - 1 year old).

What type of cardioplegia solution do you use?
- ◯ Depolarising - St. Thomas' No.2 (Plegisol)
- ◯ Depolarising - Harefield Hospital preparation
- ◯ Depolarising - blood microplegia
- ◯ Depolarising - customised
- ◯ Depolarising - other
- ◯ Hyperpolarising - Custodiol HTK
- ◯ Hyperpolarising - other
- ◯ Modified depolarising - del Nido
- ◯ Other

Customised/other - please provide details of composition including base solution, salts and additives: ____

What composition do you use?
- ◯ Crystalloid
- ◯ Blood

What ratio of blood:crystalloid do you use?
- ◯ 1:4
- ◯ 1:1
- ◯ 2:1
- ◯ 3:1
- ◯ 4:1
- ◯ Other

Other - please provide details: __________________________________

Do you add any of the following to your cardioplegia?
- ☐ Adenosine
- ☐ Lidocaine
- ☐ Magnesium sulphate
- ☐ Dextrose
- ☐ Mannitol
- ☐ Procainamide
- ☐ Other
- ☐ No additives

Other additives - please provide details: __________________________________

What is the temperature of your cardioplegia (Celsius)?
- ◯ Below 4 degrees
- ◯ 4-6 degrees
- ◯ 7-9 degrees
- ◯ 10-12 degrees
- ◯ 13-20 degrees
- ◯ 21-30 degrees
- ◯ Above 30 degrees

Do you use topical cooling of the heart eg. ice slush?
- ◯ Yes
- ◯ No
- ◯ Sometimes

Sometimes - please provide details: __________________________________

Do you give a 'hot shot', a dose of warm cardioplegia before the aortic cross-clamp is removed?
- ◯ Yes
- ◯ No
- ◯ Sometimes

Yes/sometimes - please provide details: __________________________________

What route of administration of cardioplegia do you use?

- ◯ Antegrade only
- ◯ Retrograde only
- ◯ Either antegrade or retrograde
- ◯ Combined antegrade/retrograde

Either/combined - please provide details: ______________________________

What induction dose of cardioplegia do you give?

- ◯ 5 ml/kg
- ◯ 10 ml/kg
- ◯ 15 ml/kg
- ◯ 20 ml/kg
- ◯ 25 ml/kg
- ◯ 30 ml/kg
- ◯ 35 ml/kg
- ◯ 40 ml/kg
- ◯ >40 ml/kg
- ◯ Other

Other - please provide details: ______________________________

What maintenance dose of cardioplegia do you give?

- ◯ None
- ◯ 5 ml/kg
- ◯ 10 ml/kg
- ◯ 15 ml/kg
- ◯ 20 ml/kg
- ◯ 25 ml/kg
- ◯ >25 ml/kg
- ◯ Other

Other - please provide details: ______________________________

What is your main indication for giving a maintenance dose?

- ◯ Time (even if no activity)
- ◯ Electromechanical activity
- ◯ Myocardial temperature
- ◯ Other
- ◯ None

Other - please provide details: ______________________________

What is your usual/preferred interval between doses?

- ◯ Continuous
- ◯ 10-15 minutes
- ◯ 20-25 minutes
- ◯ 30-35 minutes
- ◯ 40-45 minutes
- ◯ 50-55 minutes
- ◯ >= 60 minutes
- ◯ Single dose

Single dose - what circumstances would lead you to give a second dose? ______________________________

Now please consider your usual/current practice in patients OTHER THAN INFANTS.

Does your practice differ from what you have described above according to the age of the child?
◯ Yes
◯ No

Yes - is it different in NEONATES (< 30 days)?
◯ Yes
◯ No

Neonates - please provide details:
____

Yes - is it different in CHILDREN (1-18 years)?
◯ Yes
◯ No

Children - please provide details:
____

Does your practice vary according to the expected complexity of the repair?
◯ Yes
◯ No

Yes - please provide details:
____

Finally, we would like to assess the willingness of the paediatric cardiac surgery community to change their practice within the context of a multi-centre randomised controlled trial. The proposed trial would be designed and conducted collaboratively through the Congenital Trials Network, ideally at all centres in the UK & Ireland.

del Nido cardioplegia is the most commonly used solution in paediatric cardiac surgery in the United States. The Boston Children's Hospital protocol uses a 1:4 blood:crystalloid ratio, with a 20 ml/kg arresting dose delivered at 8-12 degrees via a recirculating system with a low-volume dead space (Matte & del Nido JECT 2012).

Would you be willing to use del Nido cardioplegia in the context of a clinical trial?
◯ Yes
◯ No

Are there any patient groups in whom you would NOT be willing to randomised to del Nido cardioplegia?
____

del Nido cardioplegia is often given as a single dose or with a longer interval between doses (60-90 minutes). What interval would you be willing to accept, assuming there is no electromechanical activity?
◯ < 45 minutes
◯ 60 minutes
◯ 90 minutes
◯ 120 minutes
◯ Other

Other - please provide details:
____

No - what are your concerns over using del Nido cardioplegia in your patients?
____

Would you need any additional equipment or disposables in order to give del Nido cardioplegia in a 1:4 blood:crystalloid preparation to neonates, infants & children?

◯ Yes
◯ No

What additional equipment/disposables would you need?

____

Would you be willing to use Custodiol HTK cardioplegia in the context of a clinical trial?

◯ Yes
◯ No

Are there any patient groups in whom you would NOT be willing to randomised to Custodiol HTK cardioplegia?

____

Custodiol HTK cardioplegia is often given as a single dose or with a longer interval between doses (60-90 minutes). What interval would you be willing to accept, assuming there is no electromechanical activity?

◯ < 45 minutes
◯ 60 minutes
◯ 90 minutes
◯ 120 minutes
◯ Other

Other - please provide details:

____

No - what are your concerns over using Custodiol HTK cardioplegia in your patients?

____

Would you be willing to use Harefield preparation in a 4:1 blood:crystalloid ratio in the context of a clinical trial?

◯ Yes
◯ No

Are there any patient groups in whom you would NOT be willing to randomised to Harefield preparation?

____

No - what are your concerns over using Harefield preparation cardioplegia in your patients?

____

Would you be willing to use St. Thomas' Hospital crystalloid solution No.2 (Plegisol) in the context of a clinical trial?

◯ Yes
◯ No

Are there any patient groups in whom you would NOT be willing to randomised to St. Thomas' Hospital solution No.2 (Plegisol)?

____

No - what are your concerns over using St. Thomas' Hospital solution No.2 (Plegisol) cardioplegia in your patients?

____

We would be most grateful for your thoughts on taking part in a multi-centre randomised controlled trial of paediatric cardioplegia in the UK & Ireland.

____
